# Integrative Taxonomy within *Eremias multiocellata* Complex (Sauria, Lacertidae) from the Western Part of Range: Evidence from Historical DNA

**DOI:** 10.3390/genes13060941

**Published:** 2022-05-25

**Authors:** Valentina F. Orlova, Evgeniya N. Solovyeva, Evgenyi A. Dunayev, Natalia B. Ananjeva

**Affiliations:** 1 Zoological Museum, Moscow State University, Bolshaya Nikitskaya 2, 125009 Moscow, Russia; val_orlova@mail.ru (V.F.O.); anolis@yandex.ru (E.N.S.); dunayeve@mail.ru (E.A.D.); 2 Zoological Institute of RAS, 199034 St. Petersburg, Russia

**Keywords:** Lacertidae, *Eremias multiocellata* complex, *E. kokshaaliensis*, taxonomy, barcode COI, central Asia

## Abstract

The Kokshaal racerunner, *Eremias kokshaaliensis* Eremchenko et Panfilov, 1999, together with other central Asian racerunner species, is included in the *Eremias multiocellata* complex. In the present work, for the first time, the results of the analysis of historical mitochondrial DNA (barcode) are presented and the taxonomic status and preliminary phylogenetic relationships within the complex are specified. We present, for the first time, the results of the molecular analysis using historical DNA recovered from specimens of several species of this complex (paratypes of the Kokshaal racerunner and historical collections of the Kashgar racerunner *E. buechneri* from Kashgaria) using DNA barcoding.

## 1. Introduction

Modern phylogenetic methods allow a look into the past with the extraction of DNA from specimens collected a century and a half ago. Collections at the Zoological Institute in St. Petersburg and Moscow State University have herpetological samples stored in ethanol that have never been exposed to formalin that go back to the 1880s. This study accesses this unique resource to answer taxonomic questions in the lizard genus *Eremias*. Currently, the genus *Eremias* includes 40 species [1] of predominantly arid sand, steppe, and desert lizards distributed from Northern China, Korea, central and southwestern Asia to southeastern Europe [2,3,4]. The central Asian racerunners of the *Eremias multiocellata—E. przewalskii* complex belong to the taxonomically most complicated group of the genus *Eremias* [5,6,7,8], which includes 8–9 central Asian species: Tien Shan racerunner *E. stummeri* Wettstein, 1940 st. nov. Eremchenko et Panfilov, 1999; Szczerbak’s racerunner *E. szczerbaki* Eremchenko et Panfilov, 1999 status nov.; Yarkand racerunner *E. yarkandensis* Blanford, 1875; Kokshaal racerunner *E. kokshaaliensis* Eremchenko et Panfilov, 1999; Kashgar racerunner *E*. *buechneri* Bedriaga, 1907; Dzhungar racerunner *E. dzhungarica* Orlova et al., 2017; multi-ocellated racerunner *E. multiocellata* Günther, 1872; Przewalski’s racerunner *E*. *przewalskii* (Strauch, 1876); *E. m.* var. *reticulata* Bedriaga, 1912 (status questionable). The genetic variability and phylogenetic relationships between *E. multiocellata, E. przewalskii* and allied taxa remain insufficiently studied. Further progress in this area is hampered by complicated taxonomy and a lack of verified identifications and comparisons with type material. Until recently, the multi-ocellated racerunner, *Eremias multiocellata,* was considered as a single species with several subspecies, only one of which, *E. m. yarkandensis,* was recognized before 1992 in the territory of the former USSR [2,5]. Long before that, an interesting population of racerunners was reported from the Sary-Dzhaz syrts of Kyrgyzstan [9], which was later assigned to the Kashgar racerunner *E. buechneri* [10]. This population, however, differs markedly from the Kashgar racerunner both in habitus and in other characteristics of external morphology [11]. As a result of many years of studying the herpetofauna of Kyrgyzstan, by applying integrative analysis to taxonomically complex groups or using integrative analysis to identify taxonomically complex groups, it was shown that racerunners from the *E. multiocellata* complex are represented by several distinct subspecies [12].

Later on, *E. stummeri* Wettstein, 1940 inhabiting the Chu-Issyk-Kul Lake basin, *E. szczerbaki* Eremchenko et Panfilov, 1992 from the inner Tien Shan and the Naryn River basin, and *E. yarkandensis* Blanford, 1875 from the Tarim basin in China were recognized as distinct species [13]. The Yarkand racerunner from the Tarim basin in China has penetrated to the territory of Kyrgyzstan (eastern Alai). *E. kokshaaliensis* Eremchenko et Panfilov, 1999 is distributed in the extreme northeast of Kyrgyzstan (Sary-Jaz and Pokrovsky Syrts) and can be easily distinguished from all previous forms by a slender habitus, a miniature head and a speckled pattern of the dorsal side of the body. The validity of these taxa was confirmed by the authors from the results of laboratory hybridization and some craniological characteristics: the presence and size of pterygoid teeth, the texture of septomaxillae and the shape of the premaxillary process [13]. All species of this complex are allopatric. The species status of the Kyrgyz and central Asian racerunners was recently confirmed by the results of molecular analysis [14]. Unfortunately, no Kokshaal racerunner specimens from Sary-Jazz were used in the analysis carried out in the cited paper. However, the data on *Eremias* sp. from the southern slopes of the central Tien Shan (China: Xinjiang, Aksu District) were analyzed and discussed.

Museum collections, especially type specimens, represent a very important source for taxonomic studies, even in the field of molecular phylogenetics. DNA degrade in historical specimens, but modern methods and special facilities allow us to obtain data from such difficult material, including successful attempts to extract DNA from formalin-fixed museum specimens [15,16,17,18]. As the historical DNA, we consider DNA isolated from old museum samples (or historical samples) following the understanding of this term in the zoological literature [19,20,21,22]. The Laboratory of Historical DNA at the Zoological Museum of the Lomonosov Moscow State University focuses on the genotyping of museum collections, especially type specimens and the specimens from the type localities, which is of crucial importance in clarifying challenging taxonomic and nomenclature issues. Previously, we successfully obtained DNA sequences for historical reptile samples: *Phrynocephalus rossicowi* (Reptilia), *Dipus saggita* (Mammalia), *Blarinella griselda* (Mammalia), *Tonkinodentus lestes* (Chilopoda), etc. [23,24,25,26]. This paper attempts to use the possibilities of studying historical DNA for the clarification of unsolved problems in this taxonomically challenging complex.

The aim of the present paper is to analyze the taxonomic status and distribution of species of the *E. multiocellata* complex based on the molecular identification of museum collections: type specimens of *E. kokshaaliensis* and historical specimens of *E. buechneri*, as well as other collections of these *Eremias* species from China and Kyrgyzstan, including those previously used in molecular analysis [14]. We also study the uniformity of the type series, which consists of specimens collected from different localities.

## 2. Materials and Methods

The materials for this study were the following museum specimens in the herpetological collections of Zoological museum of Lomonosov Moscow State University, Moscow, Russia (ZMMU), Zoological Institute of Russian Academy of Science, St. Petersburg, Russia (ZISP) and Institute of Biology and Pedologyof the National Academy of Sciences, Bishkek, Kyrgyzstan (BSI):

*E. kokshaaliensis*: 4 topotypes: ZMMUR-3302 (3 males and 1 female): Kyrgyzstan, central Tien Shan, Sary-Dhaz River at the confluence with the Inylchek River, 05.08.1967, leg. S.L. Pereshkolnik.

*E. kokshaaliensis*: 15 paratypes: BSI R-000587–000591, 000596, 000599, 000601 (8 adults: 6 females, 2 males): Kyrgyzstan, central Tien Shan, Sary-Dhaz, vicinity of Engylchek (Enylchek), 08.1986, leg. V.K. Eremchenko.

*E. kokshaaliensis*: 5 paratypes, ZISP R-8277, males: China, Xinjiang, Kara-Teke, 1889. leg. M.V. Pevzov.

*E. kokshaaliensis*: paratype ZISP R-8289, female: China, Xinjiang, Taushkan –Darya, 1891, leg. M.V. Pevzov.

*E. kokshaaliensis*: paratype ZISP 8292, female: China, Xinjiang, southern Tien Shan, 1891, leg. M.V. Pevzov

*E. kokshaaliensis*: ZMMU R-14327, female: China, Xinjiang Uygur autonomous region, Aksu district, 35 km northeast of Aksu city, 1110 m a.s.l., spurs of Talyktau mountain ridge, 23.08.2014, leg. E.A. Dunayev.

*E. kokshaaliensis*: ZMMU R-14328 (1 male, 1 female, 1 juv.): China, Xinjiang Uygur autonomous region, Aksu district, 89 km northeast of Aksu city, 1364 m a.s.l., east spurs of Pobeda Peak Mountain, 24.08.2014, leg. E.A. Dunayev.

*E. kokshaaliensis*: ZMMU R-14329 (1 subadult male), 60 km northeast of Aksu city, 1435 m a.s.l., east spurs of Pobeda Peak Mountain, 24.08.2014, leg. E.A. Dunayev.

*E. kokshaaliensis*: ZMMU R-14330 (1 adult male, 1 subadult male and 2 juveniles: China, Xinjiang Uygur autonomous region, 75 km northeast of Aksu city 1927 (1922–2040) m a.s.l., 25–26.08.2014, leg. E.A. Dunayev.

*E. buechneri*: ZISP R-9131, 2 females: China, near Tolan-Khodjha River in Kashgaria, 1890, leg. V.I. Roborovsky.

**Figure 1:**
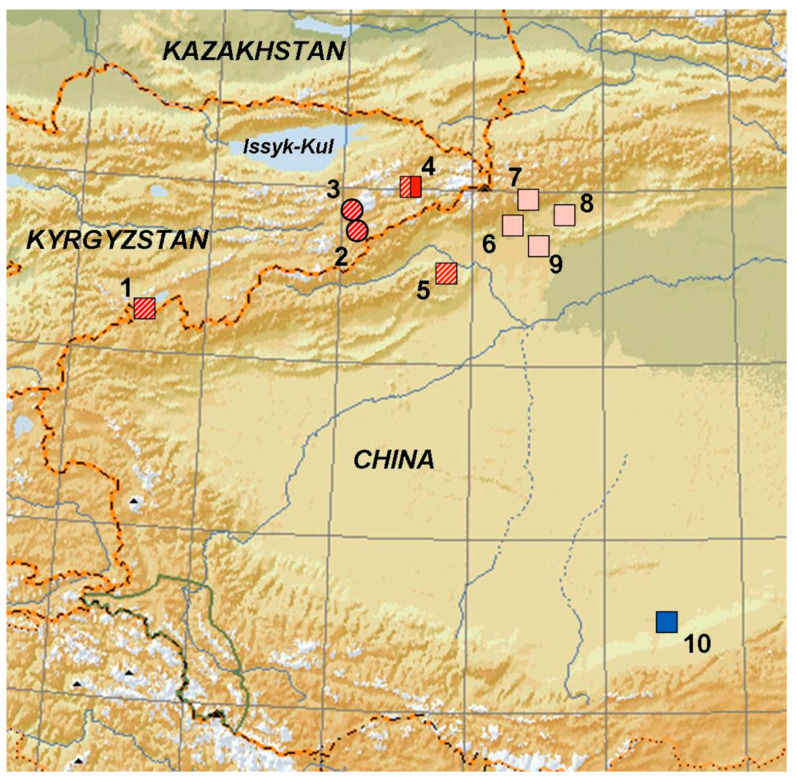
Sites of examined specimen of *Eremias kokshaaliensis* (red) and *E. buechneri* (blue). Kyrgyzstan: 1—border with China, Xinjiang, Kara-Teke (ZISP 8277); 2—Bedel valley (ZISP 8291); 3—southern Tien Shan (ZISP 8292); 4—Sary-Dhaz, vicinity of Engylchek (Enylchek (BSI R000587–591, 599–601 and ZMMU R-3302; China: 5—Taushkan–Darya (ZISP 8289); 6–60 km northeast of Aksu city, east spurs of Pobeda Peak Mountain (ZMMU 14329); 7—75 km northeast of Aksu city (ZMMU 14330); 8—9 km northeast of Aksu city, east spurs of Pobeda Peak Mountain (ZMMU 14328); 9—5 km northeast of Aksu city, spurs of Talyktau mountain (ZMMU 14327); 10—near Tolan-Khodjha River in Kashgaria (ZISP 9131). Numbers in square correspond to location of the specimens used both in morphological and molecular genetic analysis: solid square; this study: dashed square [14]. Red square corresponds to specimens used in this study; pink square ([27], Figure 1 and Appendix II). Numbers in circle correspond to location of the specimens used in morphological analysis only.

In the molecular genetic analysis, seven specimens of lizards of this complex (OK624437-OK624443) were used (Table 1; Figure 1).

**MtDNA sequencing and analyses.** Tissue samples of *Eremias* sp. were taken from seven specimens preserved in ethanol including five specimens of type series from collections of ZISP and BSI.

Molecular analysis (DNA extraction and PCR) was carried out in the ZMMU Laboratory of Historical DNA, equipped exclusively for the work with museum DNA specimens, where no previous work on fresh tissues had been performed. DNA was extracted and purified using the Qiagen QIAamp DNA MiniKit (Qiagen, Hilden, Germany) with some changes: overnight lysis step at 56 °C and two steps of elution with AE buffer, each with 50 μL of buffer and 5 min of incubation at room temperature. We amplified a fragment of the COI (cytochrome oxidase I subunit) gene of mitochondrial DNA. DNA was highly degraded, so only short fragments (100–200 bp) were obtained using a combination of internal primers designed for this study. Five primer pairs for five overlapping fragments were manually developed using Bioedit [28,29] and an alignment of *Eremias multiocellata*-complex sequences from GenBank (Table 2).

Amplification was performed in 22 μL reaction volumes containing 2 μL DNA, 4 μL of Evrogen HS-Screen mix, and 1 μL of each primer (10 pmol/μL). All stages of the extraction process included a blank as a negative control run in parallel. PCR products were visualized using a 1% agarose gel. PCR products were sequenced at the Evrogen laboratory using an ABI PRISM 3500xl sequencer with BigDye Terminator Chemistry v. 3.1 (Applied Biosystems, Foster City, CA, USA) using PCR primers.

Short fragments were first aligned using Seqman v5.06 software [27], assembled into longer ones and then aligned using BioEdit Sequence Alignment Editor 7.1.3.0 [30] with default parameters. Subsequently, the alignment including all complete sequences of studied samples was reviewed and manually revised if necessary. Obtained consensus sequences of *Eremias* ssp. were deposited in GenBank under the following accession numbers: OK624437—OK624443. Other sequences of *Eremias* were downloaded from GenBank [14,27] (Table 1). Two sequences of *E. multiocellata* (KY366658 and KY366636) and *Darevskia rudis* (MN613693) were included as outgroups. The final alignment used for the phylogenetic analyses comprised 676 bp for 52 ingroup sequences of *Eremias* spp. Mean inter- and intraspecific uncorrected genetic *p*-distances and sequence characteristics were calculated using MEGA 6.1.

Phylogenetic trees were reconstructed by Bayesian inference criteria (BI), by maximum-likelihood (ML) and maximum-parsimony (MP) methods. The optimal partitioning schemes for Bayesian inference analysis were identified using PartitionFinder software [31] using greedy search algorithm under BIC criterion: HKY + I+G for all positions together. Bayesian inference (BI) was performed using Mr Bayes v3.1.2 software [32] with two simultaneous runs, each with four chains, for 5 million generations. We checked the convergence of the runs and that the effective sample sizes (ESSs) were all above 200 by exploring the likelihood plots using TRACER v1.5 software [33]. The initial 10% of trees were discarded as burn-in. Confidence in tree topology was assessed by posterior probability (PP) [34]. The ML trees were generated using IQ tree software [35] using ultrafast bootstrap = 10,000 [36], the model was selected using ModelFinder software [37]: TPM2u + F+G4. The unweighted MP analysis was conducted in PAUP 4.0b10 software [38] with 1000 bootstrap replications. Additionally, we calculated decay indices for parsimonious tree using PRAP2 [39] under non-ratchet parameters.

## 3. Results

### 3.1. Sequence Characteristics

We obtained from 329 to 672 bp of each historical specimen of *Eremias*. Of the 676 aligned sites, 540 were found to be conserved, 130 variable and 110 parsimony-informative; the transition–transversion bias was estimated at 4.11 (all data given for ingroups only). The nucleotide frequencies on the light strand were 24.5% (A), 28.5% (T/U), 29.2% (C), and 17.8% (G). Uncorrected *p*-distances are provided in Table 3. Between-group distances vary from 1.46% to 9.57%, and within-group distances vary from 0 to 1.41%. A histogram showing the distribution of pairwise genetic *p*-distances demonstrates two gaps: at 3.0 and 6–7%, respectively (Figure 1). 

### 3.2. Molecular Phylogeny

**Figure 2:**
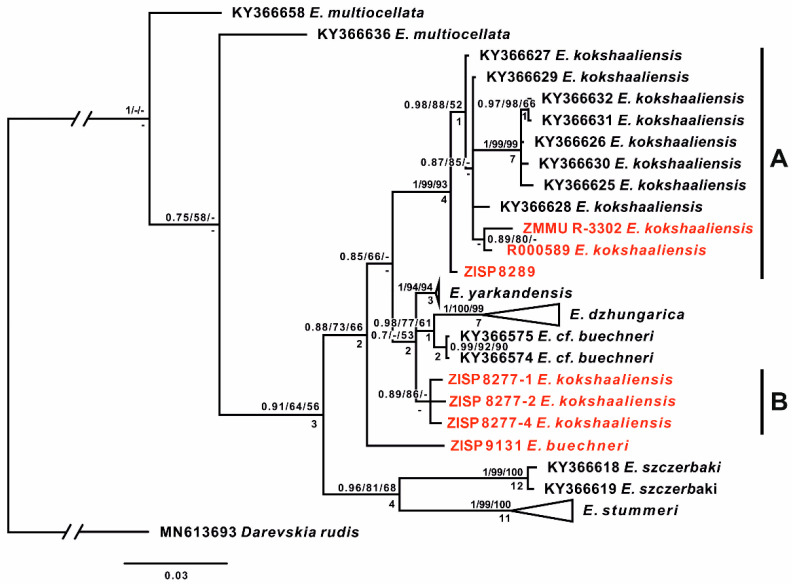
The tree resulted from the Bayesian inference analysis of the studied samples of *Eremias multiocellata* complex. Values at nodes indicate posterior probabilities/bootstrap support for BI/ML analyses, respectively. The “-” sign shows that the node exists on the Bayesian tree, but the topology is different in the specified analysis. Numbers below the nodes represent decay indices for parsimony tree. Red labels highlight sequences from historical specimens.

The extensive type series (35 specimens) of *Eremias kokschaaliensis*, selected by the authors of the description [10], includes specimens collected at different times in China and Kyrgyzstan. It includes eight historical specimens collected by M.V. Pevtsov at the end of the 19th century from Xinjiang, China (ZISP 8277, ZISP 8289, and ZISP 8291-92). In addition, it includes more recently collected specimens from Kyrgyzstan: holotype BSI R 000580 and paratypes BSI (R 000581–R000586, R 002855, Sary-Dzhaz, Terekty Gorge, 07/11/1986; R 000587–R 000591, R 000599–R 000601, Sary-Dzhaz, near the village of Engilchek (= Enilchek), 07/09/1986; R 000592–R 000595, Sary-Dzhaz, near the village of Uch-Kel, 07.09.1986; R 002005–002009, near the Sary-Dzhaz River, 08.1986; R 002834, near the Sary-Dzhaz River, 16.09.1988). We succeeded in identifying seven historical specimens, including five type specimens (BSI R000589; ZISP 8277:1, 2, 4; ZISP 8289) and one topotype from the type locality (ZMMU R-3302), as well as one specimen of *E. buechneri* (ZISP No. 9131) from Kashgaria, China. The resulting phylogeny (Figure 2) mainly agrees with the one from [14]. All studied samples of *Eremias,* excluding outgroups, form a clade (PP/BS/MP = 1/64/56) and fall into four main groups: *E. szczerbaki* (PP/BS/MP = 1/99/100), *E. stummeri* (PP/BS/MP = 1/99/100), *E. buechneri*, and part of *E. kokshaaliensis* group A (PP/BS/MP = 1/99/93). The rest of the sequences form another group with low support (PP/BS/MP = 0.7/-/53), which consists of *E. yarkandensis*, *E. dzhungarica, E.* cf. *buechneri* and the rest of *E. kokshaaliensis* (Group B).

## 4. Discussion

Our results showed that the studied samples of *Eremias kokschaaliensis* are divided into two groups. One group of *E. kokschaaliensis* A (paratype BSI no. R-000589, paratype no. ZISP R-8289, and one specimen no. R-3302 ZMMU from type territory) form a clade with *Eremias* specimens from Aksu (China) with high support (PP/BS/MP = 1/0.99/93); *E. kokshaaliensis* specimen no. R-8289 ZISP has a basal position within this group (PP/BS/MP = 0.98/0.88/52). The uncorrected within-group *p*-distance within this clade is 1.41%.

Three specimens of the type series *E. kokshaaliensis,* which are paratypes of *E. kokschaaliensis* (nos. ZISP-8277-1, ZISP 8277-2, ZISP 8277-3), form the second group: *E. kokschaaliensis* B (PP/BS/MP = 0.89/87/-). The resolution of their position is too low, and it would be impossible to assign them to any form until more molecular data have been obtained and studied.

**Figure 3:**
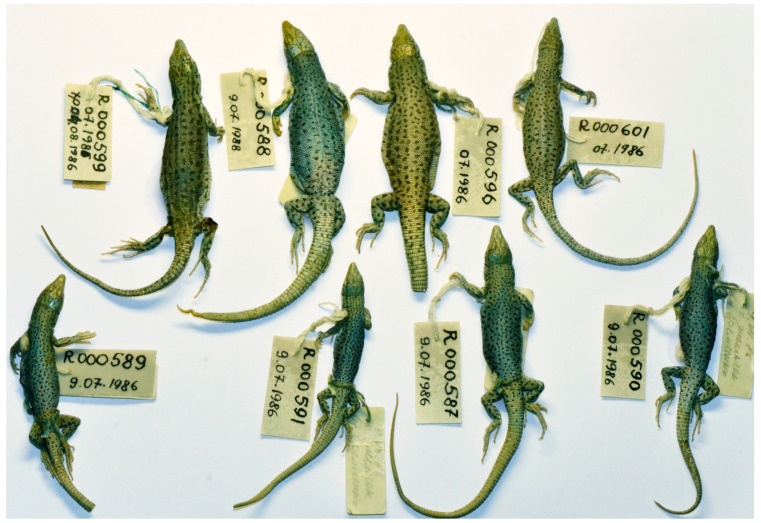
Paratypes of *Eremias kokschaaliensis* A (BSI R000587–591, 599–601) in preservative in dorsal view.

**Figure 4:**
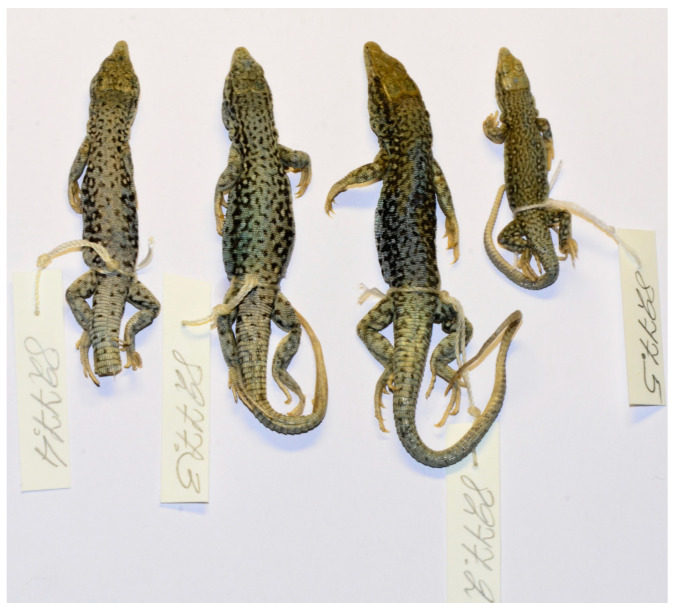
Paratypes of *Eremias kokschaaliensis* B (ZISP 8277) in preservative in dorsal view.

*E. kokshaaliensis* from the type series of group A is easily recognizable by the characteristic of the mottled pattern of the dorsal surface of the body of ethanol-preserved specimens (Figure 3 and Figure 4). As for the type specimens belonging to group B, they differ in a darker dorsolateral stripe. There may be slight differences in the number of scales around the midbody and number of subdigital lamellae on the fourth toe of the hindlimb, which is higher in *E. kokshaaliensis* from the type series of group A. It should be noted that these preliminary conclusions are not based on statistically reliable data, due to the high variability of the pholidosis characteristics and a limited number of specimens in the sample of *E. kokshaaliensis* group B. New material from the type locality of the *E. kokshaaliensis* group B is required to study the morphological variability and differences of these cryptic forms in more detail.

The *E. kokshaaliensis* group A from the Sary-Dhaz River basin occurs mainly in rocky habitats with desert vegetation, along the rocky banks of rivers and slopes of the southern, southeastern and southwestern expositions. In the area of the village of Enylchek (2500 m above sea level), it occupies sandy terraces near the river and rough-stony lower slopes of the mountains. Here they are quite common, and in one hour of the tour, there can be from 18 to 30 individuals [13]. There is no available information about the habitat of *E. kokshaaliensis* group B from Kara-Teke.

**Figure 5:**
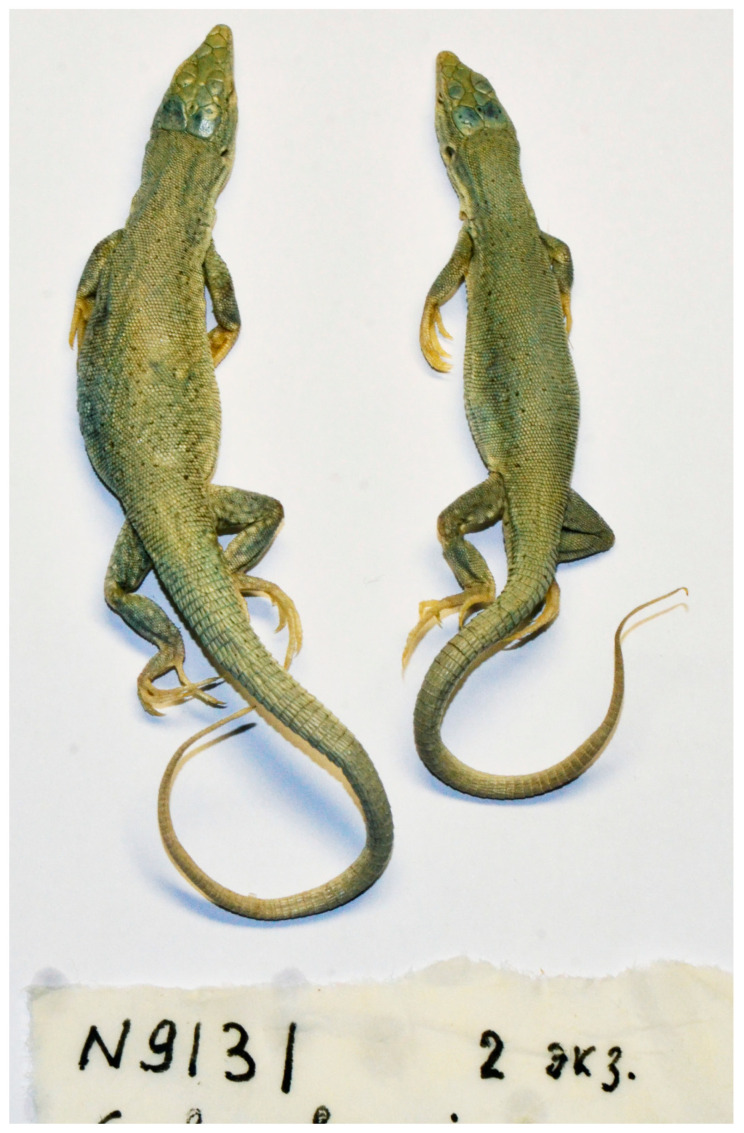
Historical specimens of *E. buechneri* (ZISP 9131) in preservative in dorsal view.

The historical specimen of *E. buechneri* no. R-9131 ZISP (Figure 5) represents a separate lineage and derives from *E. sczherbaki* and *E. stummeri*, while *Eremias* cf. *buechneri* (as mentioned in [1] groups with *E. dzhungarica* as a sister lineage (PP/BS/MP = 0.98/77/61).

It is currently not possible to discuss the phylogenetic relationships of *E. kokshaaliensis* and *E. buechneri* without newly collected specimens from Kashgaria and the results of DNA analysis.

Our DNA analysis confirmed the species designation of *E. kokschaaliensis* Eremchenko et Panfilov, 1999 and *E*. *buechneri* Bedriaga, 1907, which were previously described only on the basis of external morphology and partially on osteological and other characteristics. An important result is the revealing of the heterogeneity of the *E. kokshaaliensis* type series. The two phylogenetic groupings are geographically separated by about 300 km (Figure 1). The type of territory of the genotyped of *E. kokshaaliensis* group A coincides with the type of territory of the species, since the holotype: IBC BR 000580 originates from the same locality as the paratype BSI R000589: Kyrgyzstan, Settlement Sary-Dhaz. Thus, the phylogenetic line A corresponds to the species name given in the description, and the taxonomic status of the phylogenetic line B needs to be confirmed by new materials from the Kara Teke.

## 5. Conclusions

An important result of this study is our success in achieving the possibility of the molecular identification of historical specimens that were collected in 19th century (1880, 1881 and 1891). This novel DNA sequencing of historical specimens (ZISP) of *E. kokschaaliensis* reveals the heterogeneity of the type series and sheds light on the possibility of cryptic species within this group.

## Figures and Tables

**Table 1:** Samples used in molecular analyses.

Genbank Assession Number	Species	Specimen Voucher Number	Country	Locality	Coordinates
Sequences obtained in this study					
OK624438	*Eremias kokshaaliensis*	BSI R000589	Paratype, Kyrgyzstan	Sary-Dhaz, vicinity of Engylchek (Enylchek)	-
OK624437	*Eremias kokshaaliensis*	ZMMU R-3302	Kyrgyzstan	Kyrgyzstan, central Tien Shan, Sary-Dhaz River at the confluence with the Inylchek River	-
OK624441	*Eremias kokshaaliensis*	ZISP 8277-1	Paratype, China	China, Xinjiang, Kara-Teke,	-
OK624442	*Eremias kokshaaliensis*	ZISP 8277-2	Paratype, China	China, Xinjiang, Kara-Teke,	-
OK624443	*Eremias kokshaaliensis*	ZISP 8277-4	Paratype, China	China, Xinjiang, Kara-Teke,	-
OK624439	*Eremias kokshaaliensis*	ZISP 8289	Paratype	China, Xinjiang, Taushkan –Darya	-
OK624440	*Eremias buechneri*	ZISP 9131	China	*Kashgaria*	-
Sequences from (Orlova et al., 2017, Figure 1, Appendix II)					
KY366618	*Eremias* *szczerbaki*	ZMMU R-14342-1	Kyrgyzstan	Naryn Prov., Naryn Distr., N from Naryn	41.48 N; 75.98 E
KY366619	*Eremias* *szczerbaki*	ZMMU R-14342-2	Kyrgyzstan	Naryn Prov., Naryn Distr., N from Naryn	41.48 N; 75.98 E
KY366589	*Eremias* *stummeri*	ZMMU R-12551-1a	Kazakhstan	Almaty Prov., Rayimbek Distr., Ketmen Mts. foothills, 7–8 km E from Kegen	42.97 N; 79.32 E
KY366590	*Eremias* *stummeri*	ZMMU R-12551-2	Kazakhstan	Almaty Prov., Rayimbek Distr., Ketmen Mts. foothills, 7–8 km E from Kegen	42.97 N; 79.32 E
KY366591	*Eremias* *stummeri*	ZMMU R-12551-2a	Kazakhstan	Almaty Prov., Rayimbek Distr., Ketmen Mts. foothills, 7–8 km E from Kegen	42.97 N; 79.32 E
KY366592	*Eremias* *stummeri*	ZMMU R-12551-3	Kazakhstan	Almaty Prov., Rayimbek Distr., Ketmen Mts. foothills, 7–8 km E from Kegen	42.97 N; 79.32 E
KY366593	*Eremias* *stummeri*	ZMMU R-12551-4	Kazakhstan	Almaty Prov., Rayimbek Distr., Ketmen Mts. foothills, 7–8 km E from Kegen	42.97 N; 79.32 E
KY366594	*Eremias* *stummeri*	ZMMU R-12551-5	Kazakhstan	Almaty Prov., Rayimbek Distr., Ketmen Mts. foothills, 7–8 km E from Kegen	42.97 N; 79.32 E
KY366601	*Eremias* *stummeri*	ZMMU R-12552-1	Kazakhstan	Almaty Prov., Rayimbek Distr., central Tian Shan Mts., 15 km S from Tuzkol Lake, Zhabyrtau Mt.	42.92 N; 80.08 E
KY366604	*Eremias* *stummeri*	ZMMU R-14335-2	Kyrgyzstan	Issyk-Kul Prov., E bank of Issyk-Kul lake, env. of Karakol	42.46 N; 78.36 E
KY366607	*Eremias* *stummeri*	ZMMU R-14338-1	Kyrgyzstan	Issyk-Kul Prov., 100 km SW from Karakol, Kaji-Say env.	42.16 N; 77.18 E
KY366609	*Eremias* *stummeri*	ZMMU R-12427-1	Kyrgyzstan	Issyk-Kul Prov., NW bank of Issyk-Kul lake, 20–25 km N from Toru Aygyr, Kungei-Alatau Mts.	42.58 N; 76.41 E
KY366610	*Eremias* *stummeri*	ZMMU R-12427-2	Kyrgyzstan	Issyk-Kul Prov., NW bank of Issyk-Kul lake, 20–25 km N from Toru Aygyr, Kungei-Alatau Mts.	42.58 N; 76.41 E
KY366612	*Eremias* *stummeri*	ZMMU R-12556-2	Kyrgyzstan	Issyk-Kul Prov., env. of Balykchy, road to Akolen	42.35 N; 76.17 E
KY366613	*Eremias* *stummeri*	ZMMU R-12557-1	Kyrgyzstan	Issyk-Kul Prov., env. of Balykchy, road to Akolen	42.35 N; 76.17 E
KY366615	*Eremias* *stummeri*	ZMMU R-14339-1	Kyrgyzstan	Naryn Prov., Kochkor Distr., Kochkor	42.22 N; 75.75 E
KY366617	*Eremias* *stummeri*	ZMMU R-14341-3	Kyrgyzstan	Naryn Prov., Kochkor Distr., S from Kochkor	42.08 N; 75.66 E
KY366574	*Eremias* cf. *buechneri*	ZMMU R-8910-1a	China	Xinjiang Prov., Qarqan (Chemo) Distr., Altintag Mt., Chinbulak, 60 km S from Tura	37.51 N; 86.05 E
KY366575	*Eremias* cf. *buechneri*	ZMMU R-8910-1b	China	Xinjiang Prov., Qarqan (Chemo) Distr., Altintag Mt., Chinbulak, 60 km S from Tura	37.51 N; 86.05 E
KY366576	*Eremias dzungarica*	ZMMU R-11989-1	Kazakhstan	east Kazakhstan Prov., Aigyrkum sands, 5–7 km SW from Buran	47.98 N; 84.89 E
KY366577	*Eremias dzungarica*	ZMMU R-11989-2	Kazakhstan	east Kazakhstan Prov., Aigyrkum sands, 5–7 km SW from Buran	47.98 N; 84.89 E
KY366578	*Eremias dzungarica*	ZMMU R-12862-1	Mongolia	Khovd Aimaq, Bulgan Sum, Bayan-Mod, 11 km W from Ikher-Toli	47.06 N; 92.91 E
KY366579	*Eremias dzungarica*	ZMMU R-12862-3	Mongolia	Khovd Aimaq, Bulgan Sum, Bayan-Mod, 11 km W from Ikher-Toli	47.06 N; 92.91 E
KY366580	*Eremias dzungarica*	ZMMU R-12845-2	Mongolia	Khovd Aimaq, 7 km SW from Uyench Sum	46.93 N; 93.61 E
KY366581	*Eremias dzungarica*	ZMMU R-12845-4	Mongolia	Khovd Aimaq, 7 km SW from Uyench Sum	46.93 N; 93.61 E
KY366582	*Eremias dzungarica*	ZMMU R-12845-5	Mongolia	Khovd Aimaq, 7 km SW from Uyench Sum	46.93 N; 93.61 E
KY366583	*Eremias dzungarica*	ZMMU R-12845-6	Mongolia	Khovd Aimaq, 7 km SW from Uyench Sum	46.93 N; 93.61 E
KY366584	*Eremias dzungarica*	ZMMU R-12845-7	Mongolia	Khovd Aimaq, 7 km SW from Uyench Sum	46.93 N; 93.61 E
KY366585	*Eremias dzungarica*	ZMMU R-12845-8	Mongolia	Khovd Aimaq, 7 km SW from Uyench Sum	46.93 N; 93.61 E
KY366586	*Eremias dzungarica*	ZMMU R-12845-9	Mongolia	Khovd Aimaq, 7 km SW from Uyench Sum	46.93 N; 93.61 E
KY366587	*Eremias dzungarica*	ZMMU R-12845-10	Mongolia	Khovd Aimaq, 7 km SW from Uyench Sum	46.93 N; 93.61 E
KY366588	*Eremias dzungarica*	ZMMU R-12550-1	Mongolia	Khovd Aimaq, 24 km N from Uyench Sum	46.27 N; 92.05 E
KY366620	*Eremias yarkandensis*	ZMMU R-14344-1	Kyrgyzstan	Osh Prov., vicinity of Nura	39.65 N; 73.87 E
KY366621	*Eremias yarkandensis*	ZMMU R-14344-2	Kyrgyzstan	Osh Prov., vicinity of Nura	39.65 N; 73.87 E
KY366622	*Eremias yarkandensis*	ZMMU R-14344-3	Kyrgyzstan	Osh Prov., vicinity of Nura	39.65 N; 73.87 E
KY366623	*Eremias yarkandensis*	ZMMU R-14344-4	Kyrgyzstan	Osh Prov., vicinity of Nura	39.65 N; 73.87 E
KY366624	*Eremias yarkandensis*	ZMMU R-14344-5	Kyrgyzstan	Osh Prov., vicinity of Nura	39.65 N; 73.87 E
KY366625	*Eremias* sp.	ZMMU R-14327-1	China	Xinjiang Prov., 35 km NE from Aksu	41.40 N; 81.05 E
KY366626	*Eremias* sp.	ZMMU R-14329-1	China	Xinjiang Prov., 60 km NE from Aksu	41.54 N; 80.79 E
KY366627	*Eremias* sp.	ZMMU R-14330-1	China	Xinjiang Prov., 75 km NE from Aksu	41.74 N; 80.83 E
KY366628	*Eremias* sp.	ZMMU R-14330-2	China	Xinjiang Prov., 75 km NE from Aksu	41.74 N; 80.83 E
KY366629	*Eremias* sp.	ZMMU R-14330-3	China	Xinjiang Prov., 75 km NE from Aksu	41.74 N; 80.83 E
KY366630	*Eremias* sp.	ZMMU R-14328-1	China	Xinjiang Prov., 89 km NE from Aksu	41.56 N; 81.21 E
KY366631	*Eremias* sp.	ZMMU R-14328-2	China	Xinjiang Prov., 89 km NE from Aksu	41.56 N; 81.21 E
KY366632	*Eremias* sp.	ZMMU R-14328-3	China	Xinjiang Prov., 89 km NE from Aksu	41.56 N; 81.21 E
Outgroups					
KY366658	*Eremias multiocellata*	ZMMU R-12843-1	Mongolia	Govi-Altai Aimaq, Shargyn-Govi, 2 km SW from Khaliun Sum	45.93 N; 96.12 E
KY366636	*Eremias multiocellata*	ZMMU R-13215	China	Inner Mongolia Prov., 50 km S from Baotou	40.28 N; 110.00 E
MN613693	*Darevskia* *rudis*	DRbi86	Turkey	Cankurtaran Pass, Hopa, Artvin	41.390452 N; 41.541104 E

**Table 2:** Samples used in molecular analyses.

Primer Name	Sequence of 3′–5′	Annealing Temperature
Er.k-1aF	ACCAACCACAAAGAYATYGGCACTTT	58
Er.k-1aR	ATGAAGGCGTGGGCTGTTACGACTAC	
Er.k-2F	CCMGGCACCCTCCTAGGAGAYGAY	53
Er.k-2R	CGCCAGCTTCAACTGCTGATGATG	
Er.k-3F	GATATGGCATTYCCACGGATAAATAA	53
Er.k-3R	ACAAGTGGTGATAAAGTTRATTGCYCCTAAG	
Er.k-4F	CACGCAGGRGCATCTGTRGACCTAACTAT	63
Er.k-4R	AAGAGYATARTGATGCCGGCTGCTAGGACA	
Er.k-5F	CCCCCAAACATAACKCARTACCAAACC	56
Er.k-5R	ACTTCAGGGTGACCAAARAATCARAATAG	

**Table 3:** Uncorrected *p*-distances (%) for sequences of mtDNA COI gene for groups (below the diagonal). Values on the diagonal correspond to average uncorrected ingroup *p*-distances. Standard-error estimates are shown above the diagonal.

	*E. buechneri*	*E.* cf. *buechneri*	*E. dzhungarica*	*E. kokshaaliensis* Group A	*E. kokshaaliensis* Group B	*E. stummeri*	*E. szczerbaki*	*E. yarkandensis*	*E. yarkandensis*
***E. buechneri***	-	1.22	1.30	1.21	1.00	1.51	1.65	1.21	1.30
***E.* cf. *buechneri***	5.09	**0**	0.59	0.75	0.46	1.07	1.12	0.48	0.48
***E. dzhungarica***	6.54	2.63	**0.45**	0.81	0.70	1.14	1.18	0.68	0.73
***E. kokshaaliensis* group A**	5.70	4.36	5.45	**1.41**	0.74	1.09	1.10	0.69	0.77
***E. kokshaaliensis* group B**	3.93	1.51	3.75	4.22	**0.84**	1.06	1.11	0.50	0.53
***E. stummeri***	8.93	7.85	9.37	8.91	7.53	**1.12**	1.07	1.06	1.27
***E. szczerbaki***	9.67	8.43	9.15	8.53	8.12	7.82	**0.32**	1.11	1.31
***E. yarkandensis***	4.67	1.46	3.45	3.71	1.65	7.78	8.10	**0**	**0**

## Data Availability

Not applicable.
